# The Relationship Between Socioeconomic Status and Brain Volume in Children and Adolescents With Prenatal Alcohol Exposure

**DOI:** 10.3389/fnhum.2020.00085

**Published:** 2020-04-08

**Authors:** Kristina A. Uban, Eric Kan, Jeffrey R. Wozniak, Sarah N. Mattson, Claire D. Coles, Elizabeth R. Sowell

**Affiliations:** ^1^Public Health, University of California, Irvine, Irvine, CA, United States; ^2^Department of Pediatrics, Keck School of Medicine, Children’s Hospital Los Angeles, University of Southern California, Los Angeles, CA, United States; ^3^Department of Psychiatry, University of Minnesota, Minneapolis, MN, United States; ^4^Department of Psychology, San Diego State University, San Diego, CA, United States; ^5^Department of Psychiatry and Behavioral Sciences, Emory University, Atlanta, GA, United States

**Keywords:** brain volume, subcortical, brain development, adoption, collaborative initiative

## Abstract

The positive relationship between socioeconomic status (SES) and cognitive performance is mediated, in part, by differences in brain structure in typically developing youth. Associations between brain regions that relate to SES overlap with brain regions known to be sensitive to prenatal alcohol exposure (PAE). Animal models demonstrate that PAE attenuates neural and cognitive benefits of early life enrichment. However, whether or not environmental factors related to SES are associated with brain development in youth affected by PAE remains unknown in humans.

**Methods:**

T1-weighted magnetic resonance imaging (MRI) scans were obtained in participants with PAE and compared to age- and sex- matched Controls (*n* = 197, 48% with PAE, 44% girls, 6.5–17.7 years old). General linear modeling was utilized to examine associations between SES and subcortical brain volumes for youth with PAE compared to Controls.

**Results:**

Group by SES interactions were observed within the hippocampus (HPC), nucleus accumbens (NAc) and ventral diencephalon (vDC) (corrected *p* values <0.05), where positive associations (e.g., higher SES related to larger subcortical volumes) were observed within Controls, but not youth with PAE. *Post hoc* analyses examined associations between SES and brain volumes within each group independently, and revealed widespread positive associations among Controls (Amyg, HPC, NAc, Pallidum, Putamen, vDC), but not youth with PAE. Across both groups, larger subcortical volumes were related to higher cognitive performance.

**Conclusion:**

Typically developing youth exhibit increased subcortical volumes with increased SES, and surprisingly, this relationship is absent in adolescents with PAE. Findings suggest that subcortical brain volumes are neurocognitively relevant in both groups. The present results expand our understanding of the impact of PAE on the developing human brain within varying environmental contexts, and may inform novel environmental interventions that aim to improve, in part, on-going disruptions in brain development among youth with PAE. Our study highlights novel complexities in the pursuit to understand SES-brain associations, as we provide evidence that SES matters for brain outcomes among typically developing youth, and possibly not as much on an already altered brain as a result of PAE.

## Introduction

Individuals affected by prenatal alcohol exposure (PAE) can present with a complex profile of cognitive, behavioral, physical, and mental health problems, referred to as fetal alcohol spectrum disorder (FASD) (Abel and Hannigan, 1995; Hellemans et al., 2010). FASD is an umbrella term that includes all individuals with a PAE-related diagnosis. FASD is completely preventable, yet is in the top 3 known causes of intellectual disability. Any amount of PAE can potentially result in FASD, with a current prevalence of 2.4–4.8% in the United States (US) (May et al., 2014). Alcohol consumption among women of childbearing age varies by geography, environment and cultural practices (May et al., 2014, 2016a,b; Bakhireva et al., 2017). For example, 2017 prevalence reports of PAE in Texas ranged significantly depending on county (0 to 17.7%) (Bakhireva et al., 2017). Despite evidence that a larger proportion of women with high SES drink during pregnancy compared to mothers from low SES (Pfinder et al., 2012; McCormack et al., 2017), more severe FASD diagnoses (e.g., FAS, partial FAS) are more frequently found among mothers within lower socioeconomic status (SES) contexts (May and Gossage, 2011; Pfinder, 2014), indicating that factors relating to SES may influence developmental outcomes of PAE.

In the United States, individuals with a diagnosis under the umbrella term of FASD tend to be adopted, experience greater early life adversity, and exhibit a higher prevalence for mental health issues later in life than those without a FASD. However, there is a lack of animal and human studies that explore the divergent effects of other key environmental versus teratological effects of PAE on cognitive and brain development that occur with FASD. Interestingly, developmental outcomes associated with low SES (Hackman et al., 2010) greatly overlap with outcomes associated with FASD (e.g., stress dysregulation, low birth weight, increased mental health problems, impaired cognitive and emotional regulation) suggesting that deficits observed among individuals with FASD may extend beyond teratological effects of prenatal substance use to include factors correlated with low SES during the perinatal period.

Socioeconomic status is multifaceted, and includes components at multiple levels (e.g., individual, home, community), which makes measuring SES challenging (Hackman and Farah, 2009). Developmental outcomes relating to SES often depend on the specific component of SES being measured (Hackman and Farah, 2009). SES is most commonly measured as a combination of parental income, education and occupation, and can greatly impact access to resources to promote healthy child and adolescent development. Experiences within the environment are necessary to shape and wire the brain during development (Hensch, 2005), and PAE alters these neural sequences during various stages of perinatal development (Maier et al., 1999). Human studies have shown that trajectories of structural and functional brain development differ between youth with PAE compared to those without PAE (Lebel et al., 2012; Gautam et al., 2014, 2015a,b). Thus, we hypothesize that a combination of lasting PAE-effects plus altered neuronal responses to SES components likely interact to influence altered brain maturation among individuals with FASD. Thus, we predict SES will relate to brain outcomes differently in youth with PAE compared to youth without PAE.

Examination of the relationship between SES and cognitive functioning has been increasing over the past 4 decades, with low SES relating to delays in language, executive function, and working memory from infancy to early adulthood (for review see Hackman and Farah, 2009). However, few studies have examined underlying neural mechanisms of this positive association between SES and IQ. Recent work has shown that the long-known negative impact of low SES on cognitive and behavioral development (Noble et al., 2005) is indeed mediated by differences in brain structure during childhood in typically developing children (Noble et al., 2005, 2012, 2015; Piccolo et al., 2016). Notably, many of the cognitive outcomes (i.e., executive function, learning and memory) that are highly correlated with SES are also altered following moderate-severe PAE (Mattson and Riley, 1999; Mattson et al., 1999; Vaurio et al., 2008). However, this important aspect of the FASD experience regarding the environmental context in which the brain develops has received little attention. Thus, we aim to address this gap in knowledge in the present study by exploring brain-SES associations in youth with PAE compared to youth without PAE.

Deficits in executive functioning and working memory are consistently documented in both human and animal studies of PAE (Mattson and Riley, 1998). Human studies have demonstrated alterations in brain regions underlying these cognitive functions, including the prefrontal cortex (PFC) and hippocampus (HPC) (Astley et al., 2009; Dudek et al., 2014; Joseph et al., 2014; Donald et al., 2015). Mechanistically, animal models demonstrate an overall decrease in neuroplasticity following PAE within the PFC (Barr et al., 2005; Hamilton et al., 2010) and HPC (Uban et al., 2010; Brady et al., 2013; Fontaine et al., 2016). These same rodent models of PAE demonstrate that the neural outcomes of postnatal environmental manipulations differ in animals with PAE compared to those without PAE. For example, a partial rescue of PAE-related learning and memory deficits can be observed through environmental and early life manipulations such as neonatal handling, environmental enrichment, or motor training, and this improvement is sometimes reflected by neuroanatomical changes in the PAE animals despite lasting reductions in neural plasticity following PAE [reviewed in Hannigan et al. (2007)]. Recent animal models demonstrate the neural benefits of early life enrichment are observed but may be attenuated by PAE (Ignacio et al., 2014; Kajimoto et al., 2016), and the neural effects of early life stress may be enhanced by PAE (Comeau et al., 2014, 2015; Raineki et al., 2017). In humans, SES was associated with test performance on a response inhibition functional magnetic resonance imaging (MRI) task, but not with brain activation in youth with PAE and also in those without PAE (Ware et al., 2015). Whether or not SES is associated with brain structure in children and adolescents with PAE remains unknown.

Among recent PAE studies with adolescents, subcortical regions are perhaps more strongly implicated in a neurological signature of PAE-insult (Gautam et al., 2015a; Little and Beaulieu, 2019). Thus, here we targeted subcortical regions, with the parallel aim to extend understanding of SES-related alterations among subcortical volumes. Importantly, since the review of the present study, others have shown associations between SES factors and subcortical brain volumes among adolescents (McDermott et al., 2019; Jenkins et al., 2020; Machlin et al., 2020); therefore validating the original aim to target subcortical regions. A primary mechanism for PAE-related harm is through lasting alterations on central stress regulation (Raineki et al., 2014, 2019). With alterations in stress neurocircuitry as a potential overlapping mechanism between PAE- and SES- related effects on the developing brain (Lebel et al., 2019), we examined stress-sensitive subcortical regions: those residing within the forebrain. The present study examined differences in associations between SES and subcortical brain volume in youth with PAE compared to non-exposed youth as Controls. We hypothesize SES is positively associated with subcortical brain volumes, and that this relationship: (1) is attenuated in participants with PAE; (2) varies as a function the time from birth until adoption into permanent home; and (3) correlates with cognitive performance.

## Materials and Methods

### Participants

All procedures were approved by each site’s IRB and all subjects underwent a comprehensive informed consent/assent procedure both verbally and written. Participants were compensated with cash or gift cards for their time. High-resolution T1-weighted MRI data were obtained in children and adolescents with PAE, and their age- and sex- matched non-exposed Controls (Table 1; Total *n* = 197, 48% with PAE, 44% girls, 6.5–17.7 years old). MRI was collected from 4 imaging sites (26% Children’s Hospital Los Angeles, 31% University of Minnesota, 21% Emory, 22% San Diego State University) as part of the Collaborative Initiative on Fetal Alcohol Spectrum Disorders (CIFASD) (Mattson et al., 2010), and data collected from Phase III by 2015 was utilized in analyses. All participants were recruited through multiple sources to increase diversity, including online and print fliers in neighborhoods and community forums with a range of SES levels, as well as word of mouth from former participants. Additionally, individuals with PAE were referred through local FASD Clinics or responded to ads with local FASD-specific caregiver groups. Positive alcohol exposure histories were confirmed via review of records or maternal report and confirmed by a licensed medical doctor (Mattson et al., 2010). Participants in the PAE group were exposed to moderate to severe levels of alcohol (≥ 13 drinks/week or >4 drinks/occasion) (Mattson et al., 2010). Adoption rates differed between participants with PAE and those serving as Controls (Table 1; Boys: 4% Control, 68%. PAE; Girls: 12.5% Control, 80% PAE). General exclusion criteria included children/adolescents: (1) who were not fluent in English and/or did not learn English by age 5; (2) whose parents did not speak English; (3) who experienced claustrophobia; (4) who had metal on or in the body that could not be removed; (5) who had a vision or hearing problem that could not be corrected; (6) who thought they may be pregnant; and (7) who endorsed any previous substance use themselves (Mattson et al., 2010). In-person interviews were conducted with the primary caregiver to obtain detailed developmental histories of adolescents (including prenatal substance exposure). Control participants were excluded if they were exposed to >1 drink per week on average or >2 drinks on a single occasion during the *in utero* period (Mattson et al., 2010).

**TABLE 1:** Descriptives.

		**Control**		**PAE**	
		**Male**	**Female**	**Male**	**Female**
**General**					
	Sample Size	52	50	58	37
	Mean age at scan (years)	13.4 ± 2.5	13.5 ± 2.7	12.7 ± 2.5	12.2 ± 2.4
	Age range at scan (years)	(7.1–17.7)	(6.5–17.2)	(7.4–16.8)	(7.0–16.8)
**Site (n):**					
	Los Angeles	14	14	14	10
	Atlanta	9	12	12	11
	Minneapolis	17	13	15	10
	San Diego	12	9	15	6
**Ethnicity**					
	Hispanic or Latino	11%	18%	17%	16%
	Not Hispanic or Latino	82%	76%	70%	81%
	Unknown	5%	6%	12%	2%
**Race**					
	American Indian/Alaska Native	5%	4%	3%	13%
	Asian	13%	6%	1%	0%
	Native Hawaiian/Pacific Islander	0%	2%	1%	0%
	Black or African American	26%	38%	22%	40%
	White	67%	50%	72%	59%
	*More than one race	17%	10%	12%	16%
	Unknown	1%	6%	0%	5%
**SES Measures**					
HH Employment Value		27.8 ± 11.2	27.5 ± 11.5	27.2 ± 12.9	28.0 ± 14.1
HH Education Value		16.4 ± 3.5	16.0 ± 3.4	16.4 ± 3.2	17.3 ± 3.3
Annual Income		5.0 ± 1.7	4.6 ± 2.0	4.8 ± 1.5	5.1 ± 1.2
**Adoption Covariate**	Mean age at entry (months)	2.4 ± 11.3	3.7 ± 14.8	25.2 ± 32.4	27.6 ± 33.1
Age upon entry into permanent home	Age range at entry (months)	(0–42)	(0–48)	(0–119)	(0–108)
	% of adopted participants	4.0%	12.5%	68.0%	80.0%

Mean ± Standard Deviation, or range (low-high) when appropriate. *Individuals who identified as more than race are included in percentages for individual races.

### SES Measures

All SES measures were based on the participants’ current caregiver participating in the study. SES measures reflect the primary caregiver in the home (and not combined across all caregivers), and only SES measures at the time of participation in the study (e.g., SES measures of birth parents or historical SES of current caregivers were not assessed). Two factors (educational attainment, occupational prestige) were utilized from the Hollingshead Four-Factor Index of Socioeconomic Status to examine relationships between SES and brain volume. Educational attainment was assessed in the participating child/adolescent’s parent based on a 7-point scale assessing the highest level of education completed (e.g., 1 ≤ 7th grade; 7 = graduate/professional training). Raw parental education scores were multiplied by 3 to obtain weighted Educational Status Values for subsequent statistical analyses. Similarly, parental occupational status was based on a 9-point scale [1 = farm laborers, unskilled service workers, students, housewives (dependent on welfare, no regular occupation); 9 = higher executive, proprietor of large businesses, major professional]. Raw parental occupation scores were multiplied by 5 to obtain weighted Occupational Status Values for utilization in subsequent statistical analyses. Scores of “0” (e.g., not applicable or unknown) were not included in statistical analyses. A third measure of SES in the present study utilized the scaled assessment [from Noble et al. (2015)] to capture the current combined annual household parental income [1 = $0–9,999; 2 = $10,000–19,999; 3 = $20,000–29,999; 4 = $30,000–49,999; 5 = $50,000–74,999; 6 = $75,000–99,999; 7 = $100,000+].

### MRI Acquisition

Acquisition protocols were based on the Pediatric Imaging Neurocognition and Genetics study (PING) (Brown et al., 2012), which harmonized parameters across imaging platforms. Whole brain high-resolution structural anatomical images were acquired in the sagittal plane using a T1 weighted MPRAGE scanning sequence (CHLA: Philips Achieva v3.2.1, echo time (TE) = 3.185 ms, repetition time (TR) = 6.795, slice thickness = 1 × 1.2 × 1 mm^3^ isotropic; Emory: Siemens Trio Syngo MR B17, TE = 4.33 ms, TR = 2170, slice thickness = 1 × 1.2 × 1 mm^3^ non-isotropic; SDSU: GE Discovery MR750 DV22.0_V02_1122, TE = 2.984, TR = 7.38, slice thickness = 1 × 1.2 × 0.9 mm^3^ isotropic; UMN: Siemens Trio Syngo MR B17, TE = 4.33 ms, TR = 2170, slice thickness = 1 × 1.2 × 1 mm^3^ non-isotropic).

### MRI Preprocessing

All data were processed through FreeSurfer image analysis suite (version 5.1) recon-all automated processing pipeline, which is freely available at http://surfer.nmr.mgh.harvard.edu/). Expert neuroanatomists reviewed each output image to detect possible errors made by the program. If errors were found, manual edits were made and the image was re-processed with corrected inputs. The recon-all processing pipeline: (1) applies linear and non-linear alignments of data to a standard MNI-space template, (2) corrects for inhomogeneities in signal, (3) removes non-brain voxels from the image (skull strip), and finally (4) applies volume labeling through probabilistic atlas. 8 ROIs were selected as from the subcortical gray matter ROIs, of which the forebrain is comprised [thalamus, caudate, putamen, pallidum, hippocampus (HPC), amygdala (Amyg), nucleus accumbens (NAc), ventral diencephalon (vDC)]. Region-of-interest (ROI)-wise subcortical volumes were averaged across the left and right hemispheres for statistical analyses.

### Age at Adoption

The majority of participants with PAE lived with an adoptive family (74%; see Table 1 for details). It is possible that the associations between SES and brain measures may be attenuated among children/adolescents with PAE due to less time within the current SES context, or due to unmeasured effects of birth family SES. Thus, we utilized age (in postnatal months) upon entry into the adoptive home (e.g., 0 = not adopted, 24 = adopted at age 24 months, etc.) and age at MRI scan as covariates to begin to address this question with *post hoc* analyses to inform future studies.

### Working Memory and Executive Functioning

A secondary aim was to examine the potential cognitive relevance of observed differences in SES-brain associations between the Control and PAE groups. To do so, two cognitive functions were selected because they are pervasively altered by moderate-heavy PAE in human adolescents (Rasmussen, 2005): working memory (implicating the hippocampus) and executive functioning (implicating the prefrontal cortex and the integrated subcortical regions of the forebrain). Standardized summary scores from two subtests of the Developmental Neuropsychological Assessment (NEPSY)-II (e.g., *Delayed Spatial Memory, Inhibition Switching*) were utilized as metrics of working memory and executive functioning, respectively.

### Statistical Analyses

All analyses were conducted with the statistical program R (v3.0.2) (R Core Team, 2013). To examine associations between SES (e.g., educational value, occupational value, income) and subcortical volumes, general linear modeling (GLM) was applied, controlling for age, sex and imaging site within youth with PAE, and then within Control youth separately. False discovery rate (FDR) was utilized for multiple comparison corrections for each SES factor. Follow-up analyses for significant results passing FDR correction were conducted to replicate GLM analyses while including *age at adoption* as an additional covariate. To directly assess Group (PAE vs. Control) differences in subcortical brain volumes for significant GLM results, RMANOVA was utilized with hemisphere as within-subject measure. To confirm past cognitive findings, Group (PAE vs. Control) differences in cognitive performance were assessed with ANOVA utilizing a *Bonferroni* test (corrected *p* value = 0.025). ROIs exhibiting significant group differences in SES-brain associations were examined for potential neurocognitive relevance (e.g., hippocampus, nucleus accumbens and ventral diencephalon). GLM was used to examine group differences in brain-cognition associations for each of the 3 ROIs, and for each of the two cognitive outcomes (working memory and executive functioning). Bonferroni correction was used to control for multiple comparisons for each cognitive outcome, with a corrected *p* value of <0.016.

## Results

### Subcortical Volume

**FIGURE 1:**
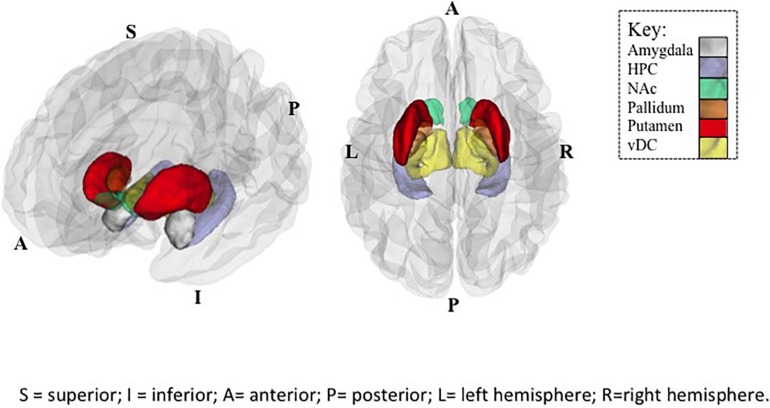
Regions of Interest (ROIs) exhibiting significant SES × subcortical volumes associations in Controls. Plotted ROIs illustrate regions where positive associations between SES and brain volumes in Controls (corrected *p* values <0.05), but not youth with PAE (n.s.) were observed in *post hoc* analyses. Regions with significant group differences in SES-volume associations include the nucleus accumbens (NAc), hippocampus (HPC), and ventral diencephalon (vDC). Image on left provides a side view, and image on right provides a top view of ROIs. Abbreviations: A = anterior; P = posterior; S = superior; I = inferior; L = left hemisphere; R = right hemisphere.

A summary of associations between SES and brain volumes can be found in Table 2 and illustrated in Figure 1. Between group analyses showed significant Group × SES interactions in the HPC (Employment (*t*(1, 197) = −2.79, *p* < 0.01) and Education (*t*(1, 197) = −2.19, *p* < 0.05)), NAc [Employment (*t*(1, 197) = −2.61, *p* < 0.01], Education [*t*(1, 197) = −2.32, *p* < 0.05)], and the vDC [Education value (*t*(1, 197) = −2.08, *p* < 0.05)]. No significant Group × SES interactions were observed with Income after FDR correction for multiple comparisons (corrected *p* values >0.24). Volumes of these subcortical regions were larger in Control compared to participants with PAE (all *p*’s < 0.01), except within the NAc (*p* = 0.78) (Table 2).

**TABLE 2:** Summary of all SES-brain associations.

**(A) Education Value**			

**ROI**	***Between-group:* Group × SES**	***Within-group:* Control**	**PAE**
Amyg	*t*(1, 197) = −1.35, *p* = 0.17	↑**	–
Caudate	*t*(1, 197) = −0.54, *p* = 0.99	↑***	–
HPC	*t*(1, 197) = −2.19, p = 0.02*		
NAc	*t*(1, 197) = −2.32, *p* = 0.02*	↑**	–
Pallidum	*t*(1, 197) = −1.38, *p* = 0.16	↑*	–
Putamen	*t*(1, 197) = −1.64, *p* = 0.10	↑*	–
Thalamus	*t*(1, 197) = −0.93, *p* = 0.09^#^	↑^#^	–
vDC	*t*(1, 197) = −2.09, *p* = 0.03*	↑**	–

**(B) Employment Value**			

Amyg	*t*(1, 197) = −0.40, *p* = 0.99	–	–
Caudate	*t*(1, 197) = −0.48, *p* = 0.99	↑**	–
HPC	*t*(1, 197) = −2.79, *p* = 0.005*		
NAc	*t*(1, 197) = −2.61, *p* = 0.009*	–	–
Pallidum	*t*(1, 197) = −1.32, *p* = 0.18	–	–
Putamen	*t*(1, 197) = −1.70, *p* = 0.09**^#^**	↑**	–
Thalamus	*t*(1, 197) = −1.32, *p* = 0.99	–	–
vDC	*t*(1, 197) = −1.80, *p* = 0.99	–	–

**(C) Income**			

Amyg	*t*(1, 197) = 0.14, *p* = 0.99	–	–
Caudate	*t*(1, 197) = 0.63, *p* = 0.99	–	–
HPC	*t*(1, 197) = –1.17, *p* = 0.24		
NAc	*t*(1, 197) = 0.003, *p* = 0.99	–	–
Pallidum	*t*(1, 197) = –0.33, *p* = 0.99	–	–
Putamen	*t*(1, 197) = –0.55, *p* = 0.58	–	–
Thalamus	*t*(1, 197) = −0.44, *p* = 0.99	–	–
vDC	*t*(1, 197) = −0.11, *p* = 0.99	–	–

**(D) Average Volumes (mm^3^)**			

Amyg**		1717 ± 345	1584 ± 331
Caudate**		3969 ± 539	3764 ± 630
HPC**		4147 ± 421	3915 ± 423
NAc		716 ± 180	723 ± 176
Pallidum**		1856 ± 261	1775 ± 289
Putamen**		6302 ± 831	6074 ± 808
Thalamus**		7591 ± 1001	7195 ± 1020
vDC**		4079 ± 514	3880 ± 518

SES-Brain Volume Associations were first examined for significant between-group differences in SES-brain associations, using 3 different SES-related factors: a) Educational Value; b) Employment Value; and c) Income. Then, within-group associations in SES-brain were examined for each group (Control only, and PAE only). ↑ indicates significant positive association; ↓ indicates significant negative association; – indicates no significant association. d) Averaged subcortical volumes across hemispheres ± standard deviation illustrate overall reduction in volume in PAE compared to Controls (** denotes all regions with *p* < 0.01) except within the NAc (*p* = 0.78). All reported *p* values corrected using FDR: ^#^corrected *p* < 0.10; *corrected *p* < 0.05; **corrected *p* < 0.01; ***corrected *p* < 0.001. C:Control; PAE: Prenatal alcohol-Exposed; Amyg: amygdala; HPC: hippocampus; NAc: nucleus accumbens; vDC: ventral diencephalon.

**FIGURE 2:**
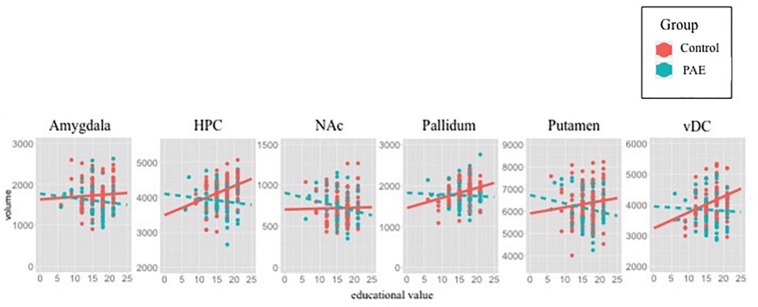
Scatterplots illustrate SES-brain associations for each ROI using Education value as SES measure and subcortical volumes (*y*-axis in mm^3^). Raw data are plotted, thus the best-fit lines are not adjusted for the utilized covariates (site, sex, race). Statistical results demonstrate positive associations between SES and volume in Control, but not PAE. The present plots with raw data corroborate statistical findings, except within the NAc, suggesting that inclusion of covariates is necessary to observe positive associations between NAc volume and SES in Controls.

Within-group analyses (e.g., Control only, PAE only) were utilized as *post hoc* examination to determine the precise nature of the significant Group × SES interactions for predicting brain volume (Figure 2). Widespread *positive* associations were observed in Control adolescents, where higher SES measures were associated with larger subcortical volumes with Education value [HPC: Employment value [*t*(102) = 2.96, *p* < 0.01], Education value [*t*(102) = 4.43, *p* < 0.0001]; NAc: Employment value [*t*(102) = 2.02, *p* < 0.05], Education value [*t*(102) = 2.88, *p* < 0.01]; vDC: Education value [*t*(102) = 2.79, *p* < 0.01] (Figure 2). However, within participants with PAE, no significant SES-brain associations were observed (FDR corrected *p* values >0.55).

### Age at Adoption

Group × SES interactions remained significant after the inclusion of “*age at adoption”* as a covariate in the HPC [Employment (*p* < 0.01) and Education (*p* < 0.05) value] and NAc [Employment (*p* = 0.055) and Education (*p* = 0.055) value], whereas the Group × SES interaction was no longer significant within the vDC [Education value (*p* = 0.47)].

### Cognitive Correlates of SES on Subcortical Volume

Only brain regions exhibiting a significant SES × Group interaction were examined to see if they are related to cognitive performance. Larger HPC volumes related to better cognitive performance across both groups HPC: working memory [*t*(195) = 2.67, *p* < 0.01], executive functioning [*t*(1, 186) = 3.01, *p* < 0.01]; and a trend within the vDC [working memory (*t*(1, 195) = 1.64, *p* = 0.10), executive functioning (*t*(1, 186) = 2.31, *p* < 0.05)]. Overall, participants with PAE exhibited lower working memory [*F*(1,189) = 20.97, *p* < 0.001)] and executive functioning [*F*(1,180) = 24.34, *p* < 0.001)] scores compared to Controls, and there were no significant group differences in brain-cognition associations in any of these regions for working memory (*p*’s > 0.21) or executive functioning (*p*’s > 0.19).

## Discussion

### Summary

Higher SES was associated with larger subcortical volumes in Controls, but not youth with PAE, within the HPC, NAc, and vDC. After controlling for the participant’s age at time of adoption, these significant Group differences in SES-brain associations remained in the HPC and NAc, but not the vDC. Overall, larger volumes of the HPC and vDC were observed in Control compared to participants with PAE, while no Group differences were observed for NAc volumes. Results within Control youth match previous findings with the hippocampus, corroborating that SES factors are important for the typically developing brain (Noble et al., 2012, 2015). Our novel results within youth with PAE suggest an absence of the typical positive relationship between SES and regional brain volumes. The SES-brain associations observed in the present study are neurocognitively important, as higher SES related to larger subcortical volumes in Controls, and larger subcortical volumes related to better working memory and executive functioning performance for both Groups.

### SES-Brain Associations Are Attenuated, or Absent, in Youth With PAE

As proposed by May et al. (2013), the effects of PAE on behavioral and cognitive outcomes are “filtered through multiple environmental conditions in the child’s formative years” (May et al., 2013). It is possible that SES-brain associations were not observed among youth with PAE as a result of reduced neuroplasticity plus increased sensitivity to stress, which could attenuate brain-SES associations. Neurobiological alterations observed in animal models of PAE demonstrate reduced capacity to benefit from protective environmental factors, as well as increased neurobiological sensitivity to harmful factors associated with low SES. Findings in the present human study corroborate these findings from animal studies.

#### Stress

In general, higher life stress is associated with lower SES (Goodman et al., 2005; Lantz et al., 2005). The subcortical regions that exhibit positive SES-volume associations in Controls but not children with PAE are known to be sensitive to stress [e.g., HPC, NAc, vDC (e.g., thalamus, hypothalamus), and putamen]. Animal studies demonstrate that PAE rats are more impacted by chronic stress in adolescence compared to controls, and become more sensitive to stress-induced cognitive deficits in adulthood (Comeau et al., 2015). It is possible that protective factors associated with high SES do not fully mitigate HPA sensitivity following PAE in human adolescents, and therefore underlies, in part, the attenuation of SES-brain associations. Among animal models, PAE results in a range of neurobiological disruptions that may underlie, in part, reduced neural responsivity to positive environmental factors (Hannigan et al., 2007). Interestingly, these alterations in neural plasticity often implicate interactive effects with underlying alterations in stress-hormone systems following PAE (Hannigan et al., 2007; Uban et al., 2010, 2013, 2015; Comeau et al., 2014).

#### Timing of Brain Development

Animal models of PAE have demonstrated a persistent reduction in neurons, microglia and oligodendrocytes. Subcortical structures were smaller in youth with PAE compared to Controls, suggesting that PAE-related alterations persist well beyond the perinatal period. Subcortical structures typically mature early relative to more prolonged maturation of lateral cortical brain regions. This relatively earlier timeline may underlie, in part, increased susceptible to prenatal, and decreased susceptibility to postnatal, factors. The present study spans a large period of brain development when many dynamic processes are occurring, including rising pubertal hormone levels. Thus, it is possible that the relationship between SES and brain volumes also exhibits a dynamic developmental trajectory, and deserves future investigation. Regardless of PAE status, larger subcortical volumes related to better cognitive performance; thus, subcortical structures may be important for future research on environmental interventions to optimize later cognitive performance in all youth.

#### Impact of SES of Birth Versus Adoptive Parents

The present study was unable to disentangle the association between SES of birth parents versus adoptive parents on brain measures. Past research shows that IQ is higher among children adopted by families with high SES compared to those adopted into low SES, and IQ is higher among children born into families of high SES compared to low SES among typically developing individuals (Capron and Duyme, 1989): suggesting unique contributions from birth- versus adoptive- family SES on cognition. Among typically developing individuals, almost half of the variability in IQ was explained by the SES of the adoptive, and not the birth, family (Capron and Duyme, 1989). However, poverty occurring earlier in life was a better predictor of cognitive performance compared to poverty experienced later in life (Duncan et al., 1998). Further, heritability of IQ is modified by SES, with higher SES contexts amplifying genetic influences (Turkheimer et al., 2003), particularly in nations where quality of education and health care vary as a function of SES like the United States (Tucker-Drob and Bates, 2016). Given that the majority of participants with PAE in the study were adopted (and very few adoptions within the Control group), this is a major limitation for interpretation of SES-brain results. Thus, whether or not the absence of SES-brain associations in the present study among youth with PAE is related to SES during perinatal development remains unknown and warrants future investigation.

### Risk Versus Protective Factors of SES

When considering the underlying mechanisms of SES-brain associations in typically developing participants, it is important to note that SES is a highly complex phenomenon, and should be viewed as an approximation of clustered health-behaviors (Pfinder, 2014). It is the differential environmental factors that are afforded across different income levels that impact brain development, rather than income level itself, *per se*. Specifically, *risk* (e.g., environmental toxins, stress) and *protective* (e.g., environmental enrichment, nutrition) factors known to be important for neural development often vary as a function of SES, where children from low SES families are generally exposed to higher levels of environmental toxins and stress/adversity, and children from high SES families are typically exposed to more environmental enrichment (Lupien et al., 2000; Morello-Frosch and Shenassa, 2006; Hackman et al., 2010; McEwen and Gianaros, 2010; Noble et al., 2012). Thus, it is possible that higher exposures to known *risk* factors for brain development may result in smaller subcortical brain volumes, and poorer cognitive performance, whereas higher exposures to known *protective* factors for brain development may result in larger subcortical brain volumes, and better cognitive performance. Given the complex nature of SES, both *risk* and *protective* factors contribute simultaneously to SES-related differences in brain development. The timing of exposure to risk or protective factors is likely unique to each family or wider community. Future research is needed to explore specific, and perhaps opposing, contributions of risk versus protective factors on brain development. The presence of cognitive differences between children from families with low compared to high SES prior to school-age suggests that SES impacts cognitive and brain development during perinatal years [reviewed in Hackman and Farah (2009)]. Further, PAE is a teratogen, which may enhance sensitivity to other environmental toxin exposures that generally occur at a higher level among low SES neighborhoods. Thus, it is possible that future studies exploring SES-brain associations that involve other toxin exposures (e.g., BPA, phthalates, air pollution, lead) may find an exaggerated, and not attenuated, relationship between SES and brain measures compared to non-exposed Controls.

### Unique Associations Among SES Factors

Interestingly, within Controls, higher educational and employment values were associated with larger volumes of several subcortical regions, but not income in the present study. Examination of different SES factors, such as education and income, might impart different risk or resilience because they may represent distinct resources that have different roles in a child’s development. Family income may provide access to material resources such as good nutrition, health care and good educational environments, whereas higher parental education in the presence or absence high family income may contribute to positive child-parent interactions (Duncan and Magnuson, 2012). Thus, it is entirely plausible that the individual factors that comprise our measurement of SES show unique, and interactive, associations with specific subcortical brain regions. Experimental manipulation of social status within the laboratory environment of a past study, elicits changes in activity within the amygdala, medial prefrontal cortex, posterior cingulate and thalamus, indicating that social status may influence development of brain regions important for processing emotional and social stimuli (Zink et al., 2008).

### Limitations of Present Study Influencing SES-Brain Associations

Several indices of SES exist that were not available for investigation in the present study, and may prove to be valuable for further elucidation of how PAE alters SES-brain associations in future studies. These include change between prenatal and adolescent SES, income-to-needs ratio (same income may afford different levels of resources across imaging sites), and other school-community based factors related to SES. Significantly more participants affected by PAE were living in adoptive homes (74%) compared to Control youth (8%); therefore SES of the *adoptive family* was analyzed in youth with PAE, but SES of the *birth family* was analyzed in Control youth. It is possible that *birth family* SES may interact with *adoptive family* SES for brain development in youth with PAE, as low SES has been previously shown to related to other known risk health-behaviors in biological mothers during pregnancy, such as greater incidence of cigarette smoking or poorer nutrition (Pfinder, 2014). Information on physical, psychological or nutritional trauma experienced between birth entry into a permanent adoptive home was not readily available in the present study, and may explain a lack of SES-brain associations among youth with PAE. A caveat to the present findings is that Figure 2 illustrates raw data and graphical best-fit lines that do not take important covariates into account (e g., sex, site, race). All regions demonstrating positive associations with SES and volume in Controls are observable in the raw data figures, except within the NAc, where the association in Controls appears to be neutral, and negative in PAE group when plotting raw data. Thus thee present findings within this region are likely dependent on incorporation of covariates, and may be difficult to replicate in future studies in the absence of these covariates. The present study focused on subcortical volumes, however, existing studies demonstrate associations between cortical measures and SES in typically developing individuals (Noble et al., 2012), and deserve further investigation among individuals with PAE.

## Conclusion

SES-brain associations in typically developing youth corroborate findings from previous research. The present study expands this understanding by revealing an absence of typical SES-brain associations following PAE. This novel finding in humans integrate well with animal literature demonstrating that PAE alters the impact of environmental factors on neurobehavioral outcomes. It is possible that reduced neural plasticity, increased stress sensitivity, prenatal SES (not measured in present study) and/or higher rates of early life adversity common to FASD may all contribute to the absence of typical SES-brain associations, warranting future investigation. The present findings expand our understanding of how PAE impacts brain outcomes in humans as a function of SES-factors. Future research on the PAE brain should investigate differential impact of risk versus protective factors that differ as a function of SES to inform interventions that aim to improve cognitive functioning among youth with FASD.

## Data Availability Statement

The datasets generated for this study will be made publicly available upon request to CIFASD. The datasets are publicly available, and all requests should be made through the Collaborative Initiative on Fetal Alcohol Spectrum Disorders (see https://cifasd.org/data-sharing/).

## Ethics Statement

The studies involving human participants were reviewed and approved by the Children’s Hospital Los Angeles, SanDiego State University, University of Minnesota and Emory’s Internal Review Boards. Written informed consent to participate in this study was provided by the participants’ legal guardian/next of kin.

## Author Contributions

KU helped design specific analytic plan, collect and analyze data, interpret and write up results, manuscript preparation and submission. EK helped collect and analyze data. JW, CC, SM, and ES helped design overall consortium under which data was collected, and provided feedback on manuscript preparation.

## Conflict of Interest

The authors declare that the research was conducted in the absence of any commercial or financial relationships that could be construed as a potential conflict of interest.
